# A transformer-based approach for early prediction of soybean yield using time-series images

**DOI:** 10.3389/fpls.2023.1173036

**Published:** 2023-06-20

**Authors:** Luning Bi, Owen Wally, Guiping Hu, Albert U. Tenuta, Yuba R. Kandel, Daren S. Mueller

**Affiliations:** ^1^ Department of Industrial and Manufacturing Systems Engineering, Iowa State University, Ames, IA, United States; ^2^ Agriculture and Agri-Food Canada, Harrow Research and Development Centre, Harrow, ON, Canada; ^3^ Ontario Ministry of Agriculture, Food and Rural Affairs, Ridgetown, ON, Canada; ^4^ Department of Plant Pathology and Microbiology, Iowa State University, Ames, IA, United States

**Keywords:** transformer, image recognition, time-series prediction, soybean yield prediction, deep learning

## Abstract

Crop yield prediction which provides critical information for management decision-making is of significant importance in precision agriculture. Traditional manual inspection and calculation are often laborious and time-consuming. For yield prediction using high-resolution images, existing methods, e.g., convolutional neural network, are challenging to model long range multi-level dependencies across image regions. This paper proposes a transformer-based approach for yield prediction using early-stage images and seed information. First, each original image is segmented into plant and soil categories. Two vision transformer (ViT) modules are designed to extract features from each category. Then a transformer module is established to deal with the time-series features. Finally, the image features and seed features are combined to estimate the yield. A case study has been conducted using a dataset that was collected during the 2020 soybean-growing seasons in Canadian fields. Compared with other baseline models, the proposed method can reduce the prediction error by more than 40%. The impact of seed information on predictions is studied both between models and within a single model. The results show that the influence of seed information varies among different plots but it is particularly important for the prediction of low yields.

## 1 Introduction

The increasing world population imposes significant challenges for agriculture production due to the increasing food demand combined with limited arable land. Accurate yield prediction can help seed companies breed for better cultivars and guide farmers to make informed management and financial decisions. However, crop yield prediction is exceptionally challenging due to several complex factors, e.g. seed type, seed treatment, soil, temperature, etc. Thus, an analytical model that can predict crop yield accurately is essential.

Machine learning methods have been designed for crop monitoring and yield prediction. Various models have been proposed for crop yield prediction. For example, Kaul et al. developed an artificial neural network model that used field-specific rainfall data and soil rating to predict soybean yield prediction (Kaul et al., 2005). Khaki et al. proposed a deep neural network approach for soybean yield prediction using genetic and environmental information (Khaki and Wang, 2019). Compared to yield prediction using meteorological driven variables (e.g., temperature, sunlight, and precipitation), using the sensing images can capture more information about the plant growing status. For example, Rembold et al. used low-resolution satellite imagery for yield prediction (Rembold et al., 2013); Nevavuori et al. presented a convolutional neural network (CNN) for crop yield prediction based on NDVI and RGB data acquired from unmanned aerial vehicles (UAVs) (Nevavuori et al., 2019); and Pantazi et al. built a hybrid model to associate the high-resolution soil sensing data with wheat yield (Pantazi et al., 2016). However, there can still be information loss in the process of using those images for yield prediction. This is because remote sensing images only provide a snapshot of the conditions at a particular moment in time, and may not capture all of the relevant factors that contribute to yield. In addition, factors such as cloud cover, shadows, and atmospheric conditions can all affect the quality and accuracy of remote-sensing images.

Compared to hyperspectral images, handheld devices capturing images of the canopy can provide higher resolution and more information due to the increased number of pixels. While higher resolution images can provide more detailed information, using data from a single time point may not be sufficient to accurately predict yield. Factors such as lighting conditions, soil status, and plant growth stage can all have a significant impact on the quality and accuracy of the image data. These undetermined factors and noise can confuse models in the training stage, resulting in the deterioration of generalization ability. The incorporation of time-series prediction is necessary for yield prediction to improve performance (Nevavuori et al., 2020; Qiao et al., 2021).

There are two challenges for yield prediction using time-series images, i.e., image processing and 48 time-series prediction. Existing studies usually use the convolutional neural network with long short term memory model (CNN-LSTM) framework for feature extraction of time-series images. For example, Sun et al. combined the CNN and LSTM to predict soybean yield using in-season and out-season image data collected from Google Earth (Sun et al., 2019). Newton et al. used 16-day remote sensing images (30m by 30m) to predict potato yield (Newton et al., 2018). Sharifi et al. applied different machine learning approaches to the barley yield prediction using the time-series NDVI and environmental information (Sharifi, 2021). However, this framework has some drawbacks.

For image classification/recognition, although the CNNs have outstanding performance on many tasks (Ferentinos, 2018; Jin et al., 2018; Ma et al., 2018), the CNNs have some redundancy issues in both computation and representations since each pixel bears varying importance for the target task. Recently, the transformer module has been considered as an alternative architecture and has achieved competitive performance on many computer vision tasks (Xie et al., 2021). Vision transformer (ViT) is a transformer-based method that is designed for image classification (Dosovitskiy et al., 2020). In ViT, an image is split into fixed-size patches. Each patch is then linearly embedded, position embeddings are added, and the resulting sequence of vectors is fed to a standard transformer encoder. Compared to CNN, ViT has a better global understanding of the images.

Regarding the time-series prediction, LSTMs have been employed to model time series in different tasks (Sundermeyer et al., 2012; Huang et al., 2015; Zhao et al., 2017). In a LSTM, the hidden state is updated with every new input token to remember the entire sequence it has seen. Theoretically, this structure can propagate over infinitely long sequences. However, in practice, due to the vanishing gradient problem, the LSTM will eventually forget earlier tokens (Li et al., 2019). Another drawback of the LSTM is that it can only be implemented sequentially due to its structure. In comparison, transformers retain direct connections to all previous timestamps, allowing information to propagate over much longer sequences and be processed in parallel.

To solve the aforementioned challenges, a transformer-based method is used to predict soybean yield using time-series images and seed information. The contribution of our work includes the following aspects:

- A method consisting of two ViT modules and one transformer is proposed for the feature extraction of time-series images. Instead of using the original images directly, the proposed method process the plant part and soil part of the image separately to reduce the computation complexity and improve the interpretability of the model.
- Different baseline models were compared to validate the effectiveness of the proposed approach. The experiments show that the proposed method can significantly improve yield prediction accuracy.
- The impact of seed information on predictions is studied both between models and within a single model. The results show that the seed information play important roles in predicting low yields.

## 2 Materials and methods

### 2.1 Data collection

**Figure 1:**
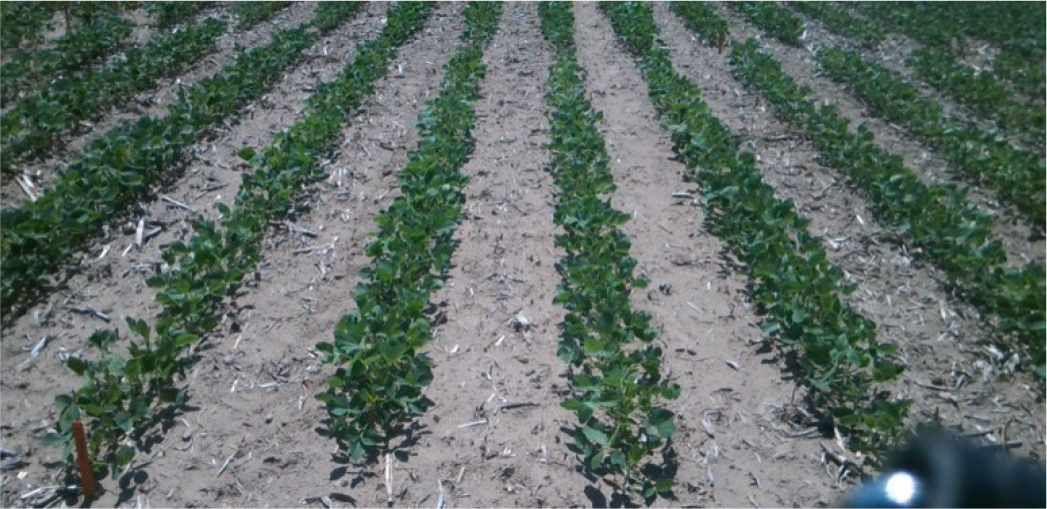
An example image of a plot.

This study used a dataset collected from three soybean fields in Ontario, Canada in 2020. There are 450 plots in total. The data includes two types of input information. The first is the time-series images. The second part is the seed information of each plot. For each plot, there are three images, as shown in **Figure 1**, collected in three dates, on June 14, 2020, on July 13, 2020 and on August 20, 2020. The seed information is shown in **Table 1**. Seed treatments are the additional material added to the seed. There are six major groups of seed treatments, i.e., Non-treated control, base seed treatment control, ILEVO alone, ILEVO+Base, Saltro+Base and other. The seed information also include seed varieties (resistant or susceptible to soybean sudden death syndrome) and seeding rates (for example, 110K, 140K, and 170K seeds/acre). The three seed factors will be investigated together. There are 51 combinations of seed varieties, treatments and seeding rates in total. The numbers of plots for each combination are similar. The objective of this paper is to use the time-series images and seed information to predict the yield.

**Table 1:** Seed information (seed treatment, seed variety and seeding rate).

Seed treatment	Number of combinations (seed variety and seeding rate)
Non-treated control	5
Base seed treatment control	9
ILEVO alone	2
ILEVO + Base	9
Saltro + Base	10
Other	16

**Figure 2:**
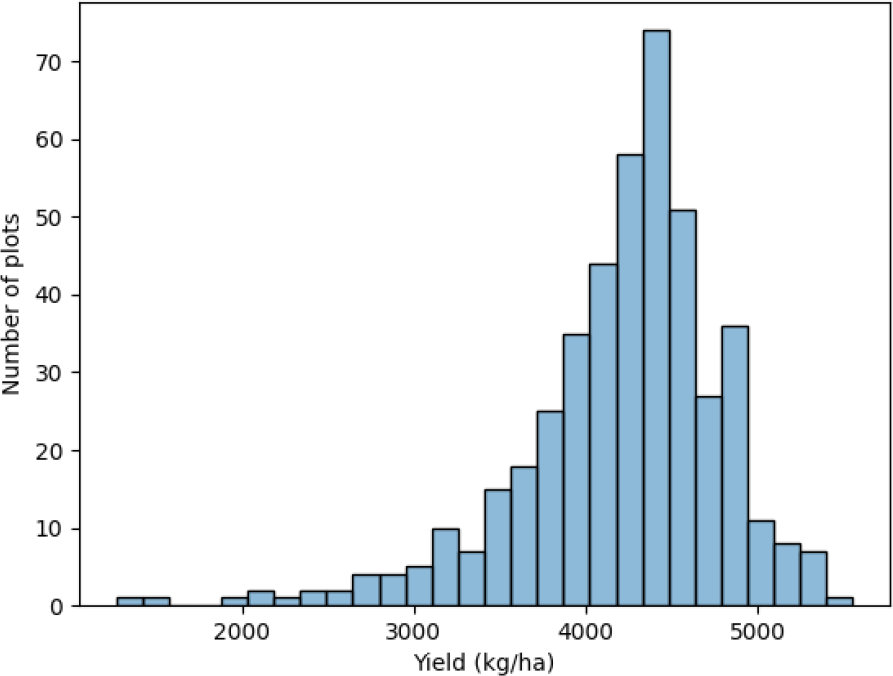
Distribution of the soybean yield.

The distribution of the yield of plots is shown in **Figure 2**. The distribution is a little left-skewed. The kurtosis is 3.09 and the skewness is -1.26. Most plots have a yield between 3500 kg/ha and 5000 kg/ha.

### 2.2 Image segmentation

**Figure 3:**
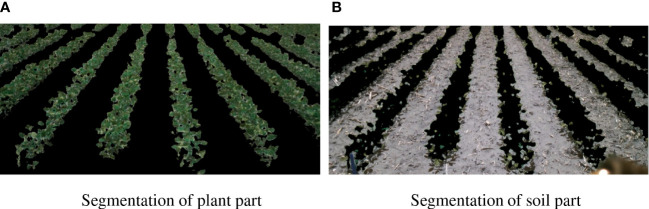
Image segmentation. **(A)** Segmentation of plant part. **(B)** This is the caption for Segmentation of soil part.

In the data processing, each image is segmented into two parts, i.e., plant segmentation and soil segmentation, as shown in **Figure 3**. This is for two reasons. First, the information extracted from plant itself with the soil can be decoupled. Each module only needs to calculate the same type of information, i.e., either plant or soil part, which will reduce the redundant computation. The interaction between plant and environment is calculated afterward. Second, it can help reduce the influence of the diagonal camera angles. The segmentation can directly tell the model the distance between two adjacent rows of plants. Thus the model can distinguish the plants at the near-end from the plants at the far end.

### 2.3 Workflow of soybean yield estimation

**Figure 4:**
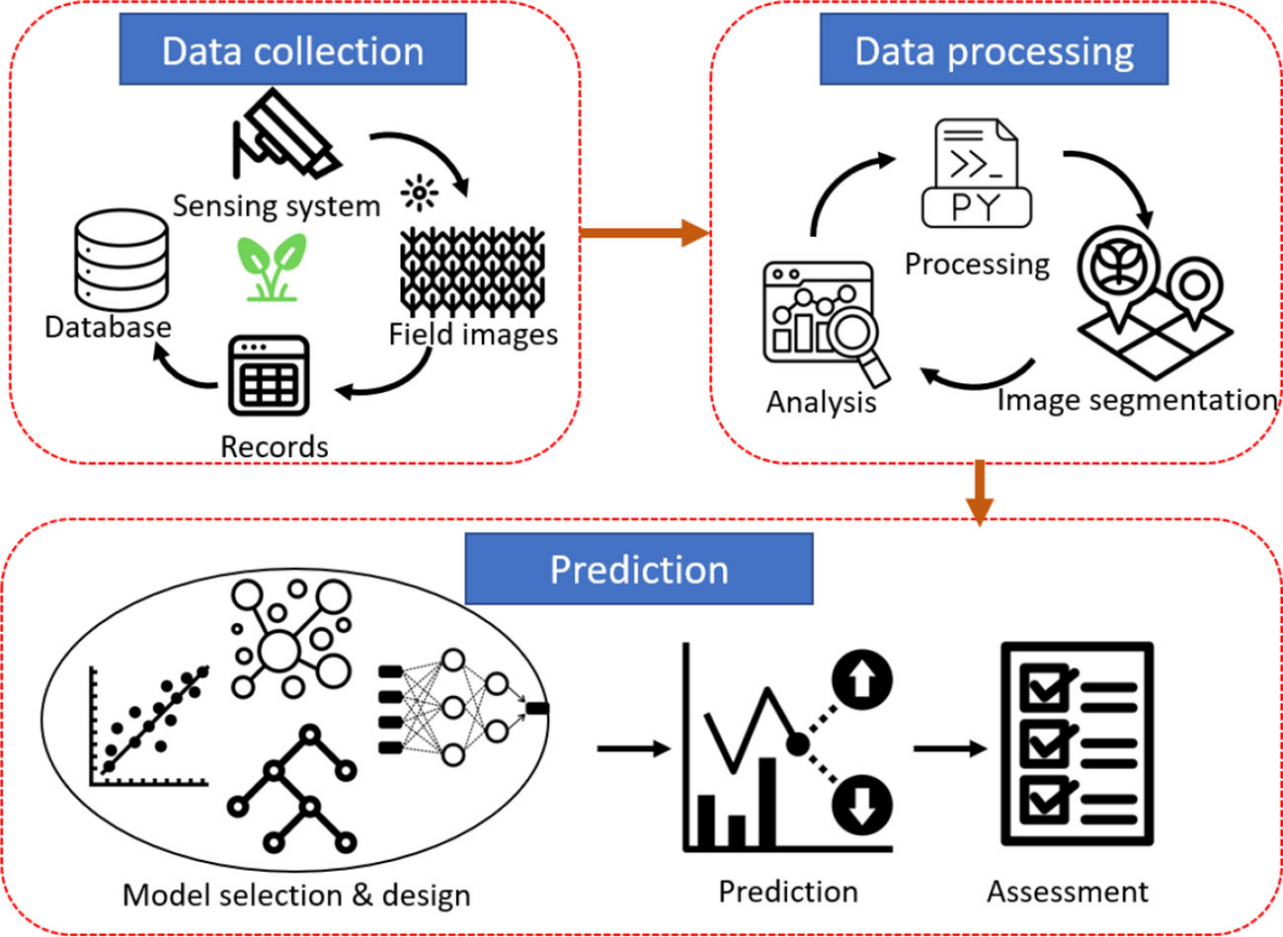
Flow diagram of the data collection, processing and prediction we employed in this study for yield prediction.

As shown in **Figure 4**, the workflow can be divided into three steps: data collection, data processing, and prediction. In the data collection, a sensing system is built to take the images of a field at a certain frequency. The images along the soybean growth stage and the checked yield are stored in the database. In data processing, some statistical analysis and image segmentation are conducted to prepare for the following analysis. Finally, various prediction models are designed to predict soybean yield. The models will be evaluated by some feasible metrics so that they can be further optimized accordingly.

The prediction is the most challenging component. The solution needs to answer three questions. How to efficiently extract features from a single image? How to detect the hidden pattern in the time-series images? How to combine different sources of information, i.e., images and seed information? This serves as the motivation of this paper.

## 3 Proposed model

To address the aforementioned challenges, a wide-deep method based on the attention mechanism is proposed. In this section, we will focus on the prediction part of the workflow, as shown in **Figure 4**, especially the design logic and module about feature extraction of the images and seed information.

### 3.1 A wide-deep framework

**Figure 5:**
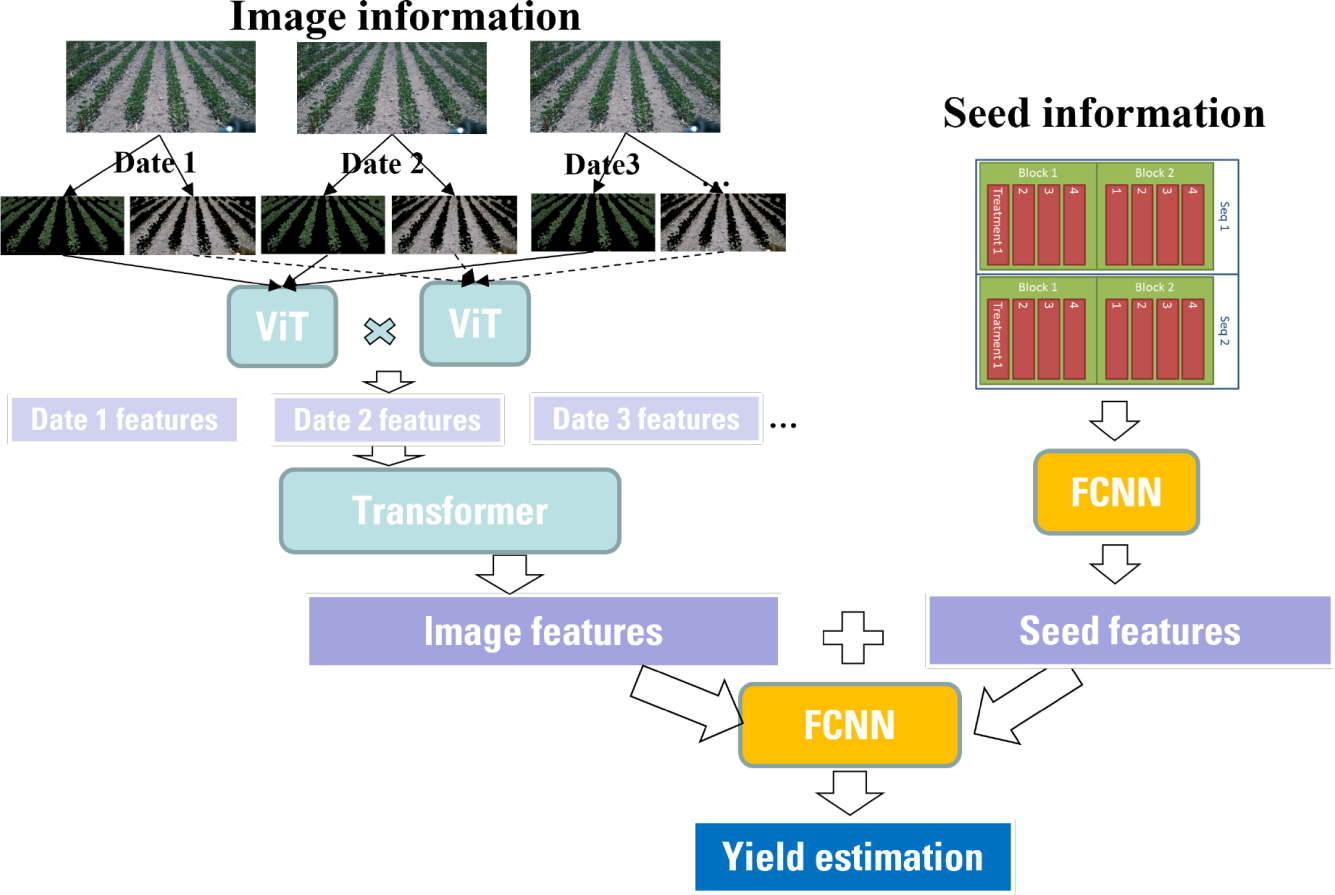
A wide-deep framework for yield prediction.

As introduced in Sec. 2, this study considers two types of inputs: time-series images and seed information. Thus, different modules should be applied due to the heterogeneity of the inputs. The time-series images have a large number of pixels. The model should be capable of extracting the most important interactions between pixels effectively. Thus, a high-level feature representation of the images is needed. In contrast, the seed information only contains one categorical variable in this study. It is not necessary to apply a complex or extremely deep neural network. Therefore, a wide-deep framework is proposed as shown in **Figure 5**.

The left tower of the proposed framework is composed of two ViT modules and one transformer module. Two ViT modules are used to extract features from the plant and soil, separately. The outputs from the two ViTs are combined using a dot product operator. Then the transformer is leveraged to deal with the time-series features. The right tower is just a fully connected neural network. The 51 combinations of seed information is one-hot encoded. Then the neural network is used to further extract information from the one-hot encoding. Finally, the wide component (i.e., seed features) and deep component (i.e., image features) are combined using one common FCDNN for joint training according to Eq. 1.


(1) $$\varphi =\text{Φ}\left(\left(f_{plant}\cdot f_{soil}\right)+f_{seed}\right)$$

Where $f_{soil}$ is the feature obtained from the soil segmentation of an image, $f_{plant}$ is the feature extracted from the plant segmentation, $f_{seed}$ is the feature extract from the seed information, i.e., seed variety, treatment and seeding rate, φ represent the predicted yield and Φ denotes a one-layer fully connected neural network (FCDNN).

It should be noted that the image features and seed features are combined and then jointly trained. This is different from ensemble train. In an ensemble model, individual models or weak estimators are trained separately without any interaction during the training process. Then their outputs are combined only at the final step (i.e., prediction) by majority voting or averaging. In contrast, the wide-deep framework will jointly train all parameters simultaneously by taking both the image features and seed features as well as the weights of their sum into account. The training of the deep-wide model is done by backpropagating the gradients from the output to both the wide and deep part of the model simultaneously using stochastic gradient descent (SGD) or other optimizers such as Adam and Adagrad. By leveraging this deep-wide framework, the training time or inference time can be significantly reduced due to fewer parameters in the wide part.

In the following sections, we will explain the details of the attention mechanism, transformer and ViT

### 3.2 Attention mechanism

Attention is a technique proposed to help the model to focus on the most important parts of its input, rather than treating all parts equally (Vaswani et al., 2017).

As shown in Eq. 2, for each input in a given vector $a_{1},a_{2},a_{3}\ldots$ , three matrices, i.e. query $W_{q}$ , key $W_{k}$ and value $W_{v}$ , are employed to generate three representation vector i.e., Q , K and V , by multiplication. Q represents the query to match other inputs. K is the key to be matched by others. V represents the information to be extracted. Then the attention score between two inputs can be calculated by Eq.3 to obtain the attention coefficients.


(2) $$Q=aW_{q},K=aW_{k},V=aW_{v}$$

(3) $$\text{Attention }(Q,K,V)=\text{softmax }\left(\frac{QK^{T}}{\sqrt{d_{k}}}\right)V$$

Where $d_{k}$ is the dimension of the keys and queries which is used to scale the dot product of Q and K Specifically, we repeat the attention for h times and concatenate the learned embeddings as the final representation of the inputs:


(4) $$\text{MultiHead }(Q,K,V)=\text{Concat }\left({\text{head}}_{1},\ldots ,\;{\text{head}}_{\text{h}}\right)W^{O}$$

Where $\;{\text{head}}_{\text{i}}=\text{Attention }\left(QW_{i}^{Q},KW_{i}^{K},VW_{i}^{V}\right)$ The attention mechanism is the backbone of transformer and ViT.

### 3.3 Vision transformer for image feature extraction

Self-Attention is capable of understanding the connection between inputs. However, it is challenging to apply it between the pixels of an image. For instance, if the size of the input image is 300x300, a self-attention layer has 90K combinations to calculate. In fact, a lot of the calculation are redundant because only part of the connections between two pixels are meaningful. To overcome this problem, ViT is proposed by segmenting images into small patches (like 16x16) (Dosovitskiy et al., 2020). A patch is the basic unit of an image instead of a pixel to efficiently tease out patterns.

In ViT, an image $x\in {\mathbb{R}}^{H\cdot W\cdot C}$ is reshaped into N patches $x_{p}\in {\mathbb{R}}^{N\cdot P^{2}\cdot C}$ , where ( H , W ) is the resolution of the original image, C is the number of channels, $P^{2}$ is the resolution of each patch. In addition to patches, ViT also use a learnable embedding $E_{pos}$ for each patch to represent the relative position. Thus, the patch embeddings can be represented as in Eq. 5.


(5) $${\text{z}}_{0}=\left[{\text{x}}_{p}^{1}\text{E};{\text{x}}_{p}^{2}\text{E};\cdots ;{\text{x}}_{p}^{N}\text{E}\right]+{\text{E}}_{\text{pos }},\;\text{E}\in {\mathbb{R}}^{\left(P^{2}\cdot C\right)\times D},{\text{E}}_{pos}\in {\mathbb{R}}^{\left(N+1\right)\times D}$$

Assuming that there are L layers in the ViT, then in each layer, multi-head attention and MLP is applied to the input of each layer as shown in Eq. 6 and Eq. 7. The calculation of multi-head attention is explained in Eq. 4.


(6) $$z_{\ell }^{'}=\text{MultiHead }\left(\text{LN }\left(z_{\ell -1}\right)\right)+z_{\ell -1},\ell =1\ldots L$$

(7) $$z_{\ell }=\text{MLP }\left(\text{LN }\left(z_{\ell }^{'}\right)\right)+z_{\ell }^{'},\ell =1\ldots L$$

Where LN is the Layernorm operator (Wang et al., 2019). LN is applied before every block, and residual connections after every block.

The last step is to output the image features as calculated using 8


(8) $$y=\text{LN}\left(z_{L}^{0}\right)$$

### 3.4 Transformer for time-series prediction

For time-series prediction, recurrent neural network (RNN) or LSTM are usually the first ones to consider. However, this type of models is hard to parallel because the models process the input of each timestamp in sequence order. Then, some studies adopted CNN to realize parallelization of the feature extraction. Nevertheless, CNN can only consider the input in a limited range. For long-term dependency modeling, CNN needs to increase the number of filters and the number of layers. Therefore, transformers based on the self-attention mechanism are applied for time-series prediction. It computes the relation between two timestamps in a bi-directional manner, which means it can be implemented in parallel.

The basic structure of a transformer used for sequence-to-sequence tasks includes encoder and decoder parts (Wu et al., 2020). Nevertheless, in this study, the task is to transform a sequence to some features. Thus, only the encoder part is used for the transformer. The encoder of the transformer is composed of an input layer, a positional encoding layer, and a stack of multi-head attention layers. The input layer maps the input time-series data to a vector through a fully-connected network. Positional encoding with sine and cosine functions is used to encode sequential information in the time series data by element-wise addition of the input vector with a positional encoding vector, which is the same as Eq. 5. Each multi-head layer is to calculate the attention coefficients between the image features of every two timestamps. Finally, there is an output layer that maps the output of the last multi-head attention layer to image features.

## 4 Baseline models and experiment settings

To validate the effectiveness of the proposed method, we compared it with other baseline models.

### 4.1 Baseline models

The three most commonly used models are implemented as the baseline models, i.e., convolutional neural network with linear regression (CNN-LR), CNN-LSTM, and vision transformer with transformer (ViT-T). The processing of seed information is the same for all baseline models and the proposed method.

#### 4.1.1 Convolutional neural network with linear regression

CNN is a class of deep, feed-forward artificial neural networks. It was adopted widely for its fast deployment and high performance on image classification tasks. CNNs are usually composed of convolutional layers, pooling layers, batch normalization layers and fully connected layers. The convolutional layers extract features from the input images whose dimensionality is then reduced by the pooling layers. Batch normalization is a technique used to normalize the previous layer by subtracting the batch mean and dividing by the batch standard deviation, which can increase the stability and improve the computation speed of the neural networks. The fully connected layers are placed near the output of the model. They act as classifiers to learn the non-linear combination of the high-level features and to make numerical predictions. Detailed descriptions on each type of function can be accessed from Gu et al. (2018).

In CNN-LR, firstly, a CNN is built to extract features from a single image. Then the obtained features from time-series images are concatenated with seed features and then used as the input of a linear regression model. Since the linear regression model cannot detect the dependency in a time series, CNN-LR is used to show the influence of time-series features.

#### 4.1.2 Convolutional neural network with long-short the memory model

Despite its popularity as a universal function approximator and easy implementation, RNN is faced with the gradient vanishing/exploding problem. In the training process of RNNs, gradients are calculated from the output layer to the first layer of the RNN. If the gradients are smaller than 1, the gradients of the first several layers will become small through many multiplications. On the contrary, they will become very large if the gradients are larger than 1. Therefore, it sometimes causes the gradients to be almost zero or very large when it reaches the first layers of RNNs. Consequently, the weights of the first layers will not get updated in the training process. Therefore, simple RNNs may not be suitable for very long time series. LSTM solves this issue by introducing the concept of gates. A common LSTM unit is composed of a cell, an input gate, an output gate and a forget gate. At each timestamp, the cell adjust its state value according to the current input and memory of previous steps. And the three gates regulate the flow of information into and out of the cell. Therefore, LSTM can extract features from long time series. Detailed explanations and calculations of each function can be accessed from Hochreiter et al (Hochreiter and Schmidhuber, 1997).

In CNN-LSTM, the first step is to extract features from a single image. Then the extracted features of images taken at different timestamps are treated as a time series. LSTM is employed to deal with the time-series features. The output obtained by LSTM is combined with seed features to get the yield prediction through a fully connected neural network.

#### 4.1.3 Vision transformer with transformer

In ViT-LSTM, the image is processed using a ViT module to get the image representation. Then the time-series image features are used as the input of the LSTM module. The yield prediction is made based on the output of the transformer and the seed features.

#### 4.1.4 Vision transformer with transformer

Different from the proposed method, in ViT-T, the image is not segmented into soil and plant parts. Thus only one ViT module is utilized to read images. Then the time-series image features are used as the input of the transformer. The yield prediction is made based on the output of the transformer and the seed features.

### 4.2 Experiment settings

The module for the seed combination information processing is the same for all baseline models. The seed combination is one-hot encoded and then connected to three dense layers of 16 neurons. Thus, the output embedding size of seed information is 16. Then the seed combination embedding is concatenated with the image embedding and the concatenated vector is connected with three dense layers of 128 neurons each, followed by a dense layer with one neuron to produce the final prediction. To avoid the overfitting issue caused by limited data, all models are using early stopping and dropout techniques. This encourages the network to learn more robust features by preventing individual nodes from becoming too specialized on a particular set of features. The dropout rate used for the dense layers is 0.25, except for the output layer which uses a linear activation function. The early stopping is used with the patience of 10 epochs. The time-series image processing part of the models is as follows.

In the CNN-LR model, the convolutional neural network (CNN) module uses the VGG-16 architecture, which consists of 13 convolutional layers and 3 fully connected layers (Simonyan and Zisserman, 2014). The linear regression module is applied with L2 norm regularization to prevent overfitting. The model expects the input to be a three-dimensional tensor of size (128, 128, 3) representing the image size and number of channels. The output of each time-series image from the VGG model is flattened and concatenated together. The concatenated image embeddings are connected with a dense layer (i.e., the LR module) of 256 neurons to extract features from the images. In CNN-LSTM, the CNN module is the same as that in CNN-LR. The output of time series images from the VGG model is processed by a LSTM module which has two bi-directional LSTM layers. Each LSTM layers contains 128 neurons. The output from the LSTM module is 128 neurons.

In the ViT-LSTM model, the Vision Transformer (ViT) module consists of two multi-head attention layers, with each layer having 3 heads. The output of the time-series images from the VGG model is then processed by same LSTM module as in CNN-LSTM. In the ViT-T model, the Vision Transformer (ViT) module consists of two multi-head attention layers, with each layer having 3 heads. The output of the time-series images from the VGG model is then processed by a transformer module, which also has 3 multi-head attention layers, each with 5 heads. The output from the transformer module is 128 neurons.

The proposed method uses two ViT modules, one to process the plant part of the image and another to process the soil part of the image separately. The ViT modules have the same architecture as that in the ViT-T model, with two multi-head attention layers, each with 3 heads, and a transformer module with 3 multi-head attention layers, each with 5 heads. The output embeddings from the plant and soil ViT modules have the same size of 128 neurons.

All models are trained using the mean squared error (MSE) loss function and the Adam optimizer with a learning rate of 0.001. 344 plots are used as the train set. 38 plots are used as the validation set. 68 plots are used as the test set.

Three metrics are used to assess the model performance, i.e., root mean squared error (RMSE), R squared value, and mean absolute error percentage (MAPE). The calculations are as in Eq. 9, Eq. 10 and Eq. 11.


(9) $$RMSE=\sqrt{\frac{1}{n}\text{Σ}{\left(y-\hat{y}\right)}^{2}}$$

(10) $$R^{2}=1-\frac{RSS}{TSS}$$

(11) $$MAPE=\frac{1}{n}\text{Σ}\left(\left|\frac{y-\hat{y}}{y}\right|\right)$$

Where n is the number of samples, y is the ground-truth yield, $\hat{y}$ is the predicted yield, RSS is the sum of squares of residuals, and TSS represents the total sum of squares.

## 5 Results

### 5.1 Comparisons with baseline models

**Figure 6:**
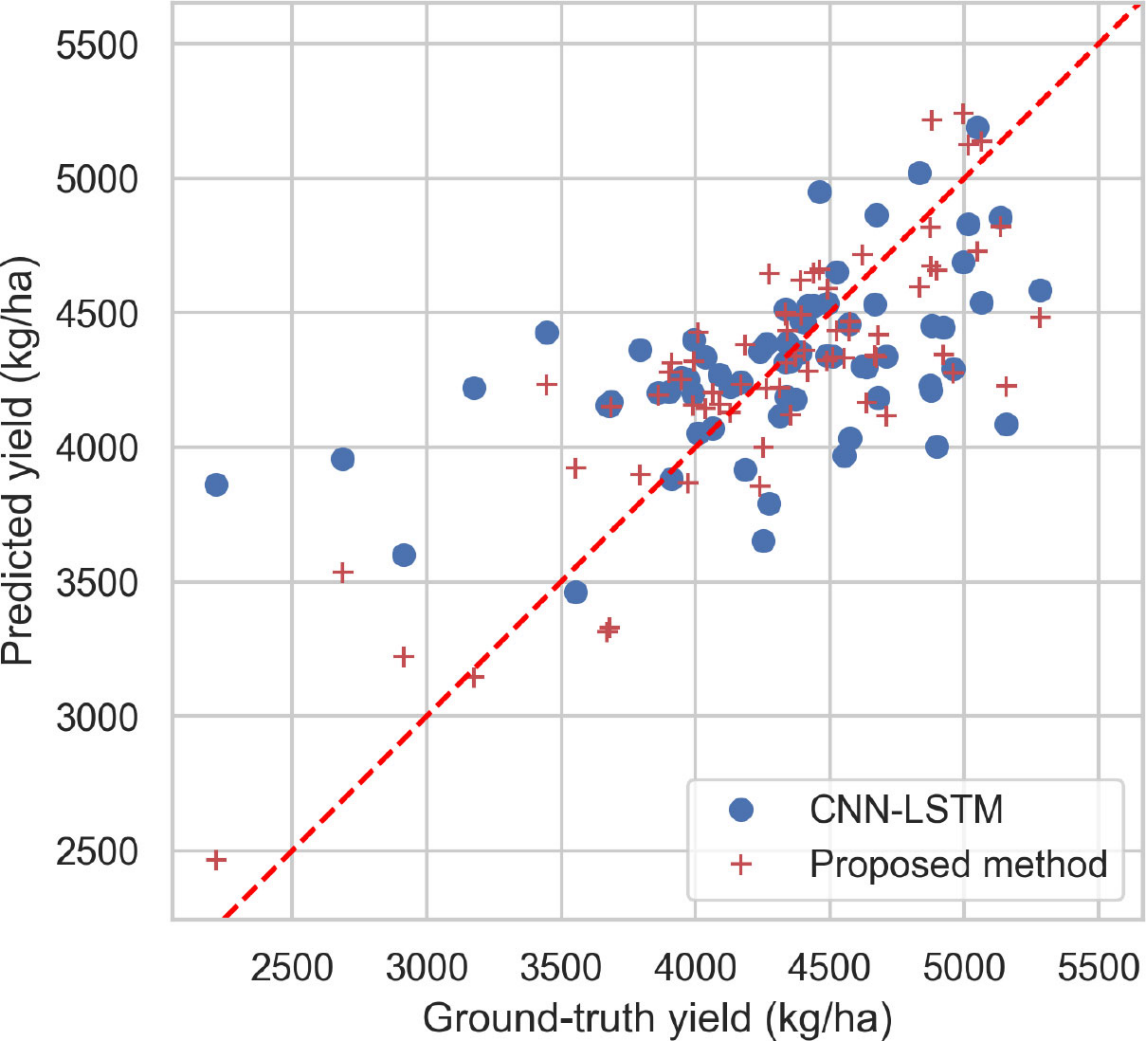
Predicted values and the ground truth for the test set.

The performance of CNN-LSTM and the proposed method are compared by plotting their predicted values and the ground truth for the test set in **Figure 6**. The results show that, in general, the predicted values of the proposed method are closer to the ground truth than those of CNN-LSTM. Moreover, it is observed that the models tend to be conservative in making predictions. For instance, in two plots where the ground truth values are between 2200 kg/ha and 3000 kg/ha, both models predict values above 3140 kg/ha. The proposed method performs better than CNN-LSTM in these two plots. Additionally, while the predicted values of CNN-LSTM was between 3900 kg/ha and 4500 kg/ha for other plots, the predicted values of the proposed method shows more diversity, indicating its ability to perform better in extreme cases.

**Table 2** presents the test RMSE, R-squared, and MAPE values obtained in this study. If the mean value of each combination of seed information is used as the estimate, the test RMSE, R-squared, and MAPE values are 570.596, 0.010, and 12.412%, respectively. The R-squared value of 0.010 indicates that using only the seed information yields slightly better results than using the mean values of all train plots. However, the introduction of CNN-LR improves the RMSE, R-squared, and MAPE by 11.7hance prediction accuracy.

**Table 2:** Comparisons between baseline models and the proposed method.

Processes	Module	Aver-seed	CNN-LR	CNN-LSTM	ViT-LSTM	ViT-T	Proposed
Image	Segmentation						✓
	CNN		✓	✓			
	ViT				✓	✓	✓
Time-series	LR		✓				
	LSTM			✓	✓		
	Transformer					✓	✓
Seed	FCNN		✓	✓	✓	✓	✓
Evaluation	Test RMSE	570.569	510.959	481.191	451.615	445.619	**332.072**
	Test R squared	0.010	0.205	0.295	0.379	0.395	**0.664**
	Test MAPE (%)	12.412	9.648	9.176	8.864	8.811	**6.340**

Aver-seed: using the mean values of each combination of seed information as the estimate."✓" means that the module is selected for the corresponding process in a specific method.

ViT-LSTM uses ViT instead of CNN for image representation, which improves the RMSE by 6.2%, R-squared by 0.08, and MAPE by 0.3%, respectively. ViT-T is an upgraded version based on the CNN-LSTM structure with a multi-head self-attention mechanism, resulting in an 8.9% reduction in RMSE, a 0.1 increase in R-squared, and a 0.4% decrease in MAPE, respectively. However, the improvement of ViT-T compared to ViT-LSTM is not significant, possibly because of the short time series used in this study.

The proposed method, which includes two ViT modules and one transformer, significantly reduces the RMSE by 34.0%, increases the R-squared by 0.27, and reduces the MAPE by 2.5%. This indicates that the proposed model outperforms the other models and effectively captures the temporal and spatial dependencies in the data.

To validate the effectiveness of deep learning models in feature representation of images, this study conducts experiments on three linear regression-based models, namely Model 1, Model 2, and Model 3, using different input configurations. As presented in **Table 3**, Model 1 solely utilizes the one-hot encoded seed combination as the input, while Model 2 takes the latest image (i.e., the last image in the time series) and the one-hot encoded seed combination as input. Model 3, on the other hand, utilizes the time-series images and the one-hot encoded seed combination as input. In each model, all inputs are concatenated into one-dimensional vectors. Both Model 2 and Model 3 are linear regression models with the L2 norm regularization technique to prevent overfitting.

**Table 3:** Comparison of three linear regression-based models.

Processes	Module	Model 1	Model 2	Model 3
Image	LR		✓	✓
Time series	LR			✓
Seed	LR	✓	✓	✓
	Test RMSE	665.729	545.619	530.489
Evaluation	Test R squared	-0.348	0.094	0.144
	Test MAPE (%)	13.346	10.993	10.816

The “Image” row indicates whether image information is used as input, where the RGB (Red, Green, and Blue) values of each pixel are averaged, and the images are converted into one-dimensional vectors. The “Time series” row indicates whether time-series images are used as input. The “Seed” row indicates whether the one-hot encoded seed combination is used as input."✓" means that the module is selected for the corresponding process in a specific method.

The evaluation results reveal that Model 1 exhibits the poorest performance with a test RMSE of 665.729, a Test R-squared of -0.348, and a test MAPE of 13.346%. This performance is attributed to underfitting, which occurs when using only one input feature. By incorporating image data, Model 2 outperforms Model 1 and the Aver-seed method in **Table 2**, achieving a 3.0% improvement in RMSE and a 2.3% improvement in MAPE. Moreover, Model 3 further enhances performance by including time-series images, resulting in a 2.7% reduction in RMSE, a 0.05 improvement in R-squared, and a 0.18% improvement in MAPE. Therefore, even a simple generalized model can benefit from time-series prediction to improve performance. However, the performance of the linear regression-based models is significantly lower than that of the deep learning models presented in **Table 2**. This discrepancy is primarily due to two reasons. Firstly, the use of average RGB values may lead to significant information loss. Secondly, linear regression cannot extract the region-level features from the extensive pixel information like CNN or ViT. Hence, the results prove the importance of using large computer vision models for image processing in agriculture, which is a crucial area for future research on large datasets for various agricultural tasks such as disease detection, yield prediction, and plant status monitoring.

### 5.2 Influence of seed information

The influence of seed information (i.e., seed variety, treatment and seeding rate) on the model’s overall performance is also investigated. As shown in **Table 4**, four methods, i.e., average of all, average with seed information, proposed method without seed information, and the proposed method, are tested. Compared to using the mean values of all training samples as the estimate, using the mean values of each group can reduce the test RMSE from 585.127 to 580.59. However, the test MAPE of average with seed information is higher, which indicates that using the average with seed information only performs better in reducing the variance of the error. The improvement in R squared is 0.01. Compared to the proposed method without seed information, the proposed method can improve the RMSE by 13.1% and R squared by 0.11, respectively. It shows that using the neural network to process the seed information is more effective than just using the group average values. Besides, the model’s prediction accuracy relies more on the image information rather than the seed information.

**Table 4:** Influence of seed information.

Methods	Test RMSE	Test R squared	Test MAPE (%)
Average of all	585.127	0	11.385
Average with seed information	570.569	0.010	12.412
Proposed method without seed information	382.820	0.554	7.895
**Proposed method**	**332.072**	**0.664**	**6.340**

Average of all: using the mean values of all training samples as the estimate. Average with seed information: using the mean values of each group as the estimate.

**Figure 7:**
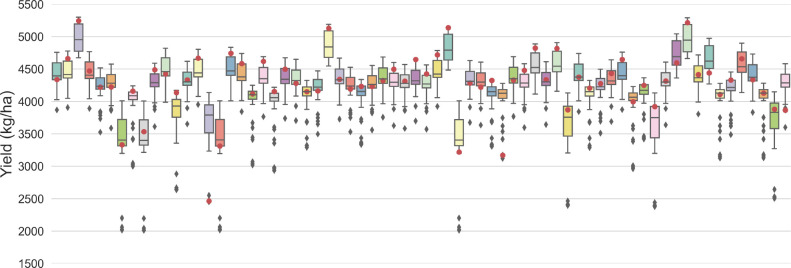
Box plot of predictions using images of each plot with 51 combinations of seed variety, treatment and seeding rate. The red dots represent the true predictions which are predicted values using the images with the true seed combination. The box plot is the result of predicted values using the images with all 51 combinations, including 50 pseudo and one true combination. The 68 test plots are numbered from 0 to 67. (Note: The true prediction is not the ground truth.).

To determine the effect of various seed variety, treatment and seeding rate on yield prediction quantitatively, an experiment is conducted by taking images of each test plot with all 51 combinations (i.e., 50 pseudo and one true combination) as the input. Thus, there are 51 predicted values for each test plot. The box plot is shown in **Figure 7**. The median value of a box can be used as an approximation for the prediction made using only images. The results, shown in a box plot, have interquartile ranges (IQRs) from 120kg/ha to 500kg/ha. A shorter IQR indicates the model extracts more information from the images, indicating its robustness to variations in seed information. This is because images taken at different stages of growth may contain additional information about the seed variety, treatment and seeding rate used. In other words, the image may contain some information that overlaps with the seed information. Comparing the true prediction with the box plot, 47 of 68 (i.e., 69.1%) test plots have true predictions within the boxes (i.e., between the 25 percentile and 75 percentile) while 21 of 68 (i.e., 30.9%) test plots fall outside the IQRs. It means the error is within 120kg/ha to 500kg/ha when replacing the true seed information with 50% pseudo combinations as the input for the test plots.

For Plot 14, 37, 41 and 67, the true predictions are outliers (below 1.5*IQR from the lower percentile) compared to all predictions, indicating the model extracts more information from the seed information for these plots. The details of Plot 14, 37, 41 and 67 are shown in **Table 5**. Plots 14, 37, and 41 have the lowest ground truth and predicted yield, but this does not necessarily indicate that “Saltro” treatments result in lower yields. It may simply mean that the model requires more information about the seed treatment to improve its predictions for certain plots.

**Table 5:** Analysis of predicted values for Plot 14, 37, 41 and 67. (Unit: kg/ha).

Plot No.	True seed information	Ground truth	True prediction	Box median value
14	Saltro: Resistant	2216.443	2470.862	3796.238
37	Saltro: Susceptible	2914.385	3219.396	3406.339
41	Saltro: Resistant	3174.018	3223.063	4140.596
67	Non-treated control: Resistant: 110K	3972.710	3864.384	4285.763

It is also worth noting that for all plots, the outlier values are below the boxes, suggesting that seed information plays an important role in helping the model predict low yields with downward correction.

## 6 Discussion

Crop yield prediction help farmers estimate yield before a field is harvested. Additionally, it can serve as an essential tool for the decision-makers to make plans regarding food security. However, many factors both genetic and environmental, before and during the season, make it challenging to obtain an accurate prediction.

Yield prediction using images recently became a popular topic due to two reasons. The first reason is that images can store all the phenotype information of the plant as well as some environmental information (i.e., soil color, light condition, etc.). The second reason is that the development of deep learning techniques in computer vision has facilitated information extraction from plant-level or field-level images. Different from the research using satellite (Rembold et al., 2013; Schwalbert et al., 2020) or UAV (Zhou et al., 2017; Hassan et al., 2019) images, this study used high-resolution camera images of field level. This will help to improve the prediction accuracy since more pixels represent more information about the plant.

Instead of using individual static imagery, the proposed framework leverages the time-series images for yield prediction. The time-series images can monitor the plant status of plants at different time points and eliminate the influence of noise on the model performance. This has been supported by many researches (Clevers, 1997; Aghighi et al., 2018; Varela et al., 2021). In our case study, the single image method, i.e., CNN-LR, is compared with the time-series image method, i.e., CNN-LSTM. The results show that time-series images can help improve test RMSE by 6.2%, R squared by 0.9%, and MAPE by 0.5%. Since each plot only has about three images, the improvement could be more significant if additional images were provided. Besides, the traditional CNN-LSTM framework (Sun et al., 2019; Nassar et al., 2020) is upgraded to the ViT-T framework by introducing the attention mechanism. CNNs are efficient in image information extraction compared to fully connected neural networks due to shared kernel weights. However, CNNs only aggregate the global information in high-level layers. ViTs incorporate more global information than CNNs at lower layers, leading to quantitatively different image features. In terms of time-series prediction, although LSTM can capture the long-term dependencies of the time series, it get inputs in sequence and cannot be implemented in parallel. Thus, ViT-T is better in the global understanding of images, computation efficiency and parallel implementation. In our case, the images were taken from one side of the plot. The information density of the image in the far end and the near end are different. Since ViT segments images into small patches, it can assign different weights according to the region/patch and achieve better granularity. The comparison results show improvements of 8.9% in test RMSE, 0.1 in R squared and 0.3% in MAPE.

Besides, the proposed method segmented the image into the plant part and the soil part. By using two ViT modules, the plant status and the environmental influence can be modeled separately. Then the two parts are multiplied to obtain soybean yield. Compared to the one-ViT version, i.e., ViT-T, the proposed method significantly reduces RMSE by 34.0%, increases R square by 0.27 and reduces MAPE by 2.5%.

Another contribution of our work is the examination of the effect of seed variety, treatment and seeding rate on predictions, both across different models and within a single model. The results of the group average method indicate that the statistical importance of seed information is limited, as the test R squared is only 0.01. However, in the proposed method, seed information contributes 0.11 to R squared compared to using the same structure without seed information input. It means the neural network can extract more information from the seed information and combine it with the image features to make predictions. The examination of the effect of seed information within the proposed method reveals that the influence of seed treatments varies among different plots. Seed treatment information is particularly important for the prediction of low yields. Additionally, the wide-deep framework can be used to incorporate more types of input information, such as genetic information, in the future.

## 7 Conclusions

Yield prediction can provide more guidelines for farmers to decide on the management plan. The development of deep learning techniques has facilitated the application of sensing techniques in precision agriculture through various types of imagery. In this study, in order to catch more global interactions between image patches and timestamps, a transformer-based method is proposed to extract image information and time-series changes of soybean status. Besides, the original images are segmented into the plant part and soil parts. A wide-deep structure is adopted to incorporate other information, i.e., seed information, into prediction. Compared to other baseline models, the proposed model can reduce the RMSE by up to 40%. The effect of seed information on predictions, both across different models and within a single model, is also examined.

This study demonstrates the potential of a large-scale computer vision model for predicting crop yield using high-resolution time-series images captured by a hand-held device. However, certain limitations must be acknowledged. Firstly, the impact of time-series length on prediction accuracy remains unexplored due to the limited size of the dataset. While it is reasonable to expect that increasing the number of images during the growth stages for training would improve the model’s performance, redundant information may also impact the model’s generalization ability or require increased computation resources. Therefore, exploring these factors’ trade-offs is a meaningful avenue for future research. Secondly, the model’s input only considers limited seed information, but could potentially benefit from the incorporation of additional information such as genetic or soil characteristics. Finally, while the attention score for images and time series is calculated separately in this study, exploring the attention score between image patches at different timestamps may improve model performance.

Despite these limitations, the proposed large-scale computer vision model demonstrates the potential for extension to various applications critical to precision agriculture, including but not limited to, disease and pest detection, weed detection and control, and crop quality assessment. These tasks require sophisticated models capable of capturing fine-grained details in plant leaves and other relevant features. Addressing the aforementioned limitations and developing more efficient multi-modal methods for yield prediction using images and environmental information represent promising avenues for future research.

## Data availability statement

The original contributions presented in the study are included in the article/supplementary material. Further inquiries can be directed to the corresponding author.

## Author contributions

LB, GH, YK, and DM conceived the idea, LB implemented the algorithm, OW, AT, YK, and DM provided the data, all reviewed the manuscript. All authors contributed to the article and approved the submitted version.
